# Development of a Web-Based System for Exploring Cancer Risk With Long-term Use of Drugs: Logistic Regression Approach

**DOI:** 10.2196/21401

**Published:** 2021-02-15

**Authors:** Hsuan-Chia Yang, Md Mohaimenul Islam, Phung Anh Alex Nguyen, Ching-Huan Wang, Tahmina Nasrin Poly, Chih-Wei Huang, Yu-Chuan Jack Li

**Affiliations:** 1 Graduate Institute of Biomedical Informatics College of Medical Science and Technology Taipei Medical University Taipei Taiwan; 2 International Center for Health Information Technology Taipei Medical University Taipei Taiwan; 3 Research Center of Big Data and Meta-analysis Wan Fang Hospital Taipei Medical University Taipei Taiwan; 4 Department of Dermatology Wan Fang Hospital Taipei Medical University Taipei Taiwan; 5 TMU Research Center of Cancer Translational Medicine Taipei Medical University Taipei Taiwan

**Keywords:** cancer, risk, prevention, chemoprevention, long-term–use drugs, drug, epidemiology, temporal model, modeling, web-based system

## Abstract

**Background:**

Existing epidemiological evidence regarding the association between the long-term use of drugs and cancer risk remains controversial.

**Objective:**

We aimed to have a comprehensive view of the cancer risk of the long-term use of drugs.

**Methods:**

A nationwide population-based, nested, case-control study was conducted within the National Health Insurance Research Database sample cohort of 1999 to 2013 in Taiwan. We identified cases in adults aged 20 years and older who were receiving treatment for at least two months before the index date. We randomly selected control patients from the patients without a cancer diagnosis during the 15 years (1999-2013) of the study period. Case and control patients were matched 1:4 based on age, sex, and visit date. Conditional logistic regression was used to estimate the association between drug exposure and cancer risk by adjusting potential confounders such as drugs and comorbidities.

**Results:**

There were 79,245 cancer cases and 316,980 matched controls included in this study. Of the 45,368 associations, there were 2419, 1302, 662, and 366 associations found statistically significant at a level of *P*<.05, *P*<.01, *P*<.001, and *P*<.0001, respectively. Benzodiazepine derivatives were associated with an increased risk of brain cancer (adjusted odds ratio [AOR] 1.379, 95% CI 1.138-1.670; *P*=.001). Statins were associated with a reduced risk of liver cancer (AOR 0.470, 95% CI 0.426-0.517; *P*<.0001) and gastric cancer (AOR 0.781, 95% CI 0.678-0.900; *P*<.001). Our web-based system, which collected comprehensive data of associations, contained 2 domains: (1) the drug and cancer association page and (2) the overview page.

**Conclusions:**

Our web-based system provides an overview of comprehensive quantified data of drug-cancer associations. With all the quantified data visualized, the system is expected to facilitate further research on cancer risk and prevention, potentially serving as a stepping-stone to consulting and exploring associations between the long-term use of drugs and cancer risk.

## Introduction

In recent decades, the prevalence of chronic medical conditions such as arthritis, osteoporosis, diabetes, hypertension, and cardiovascular disease has increased [1]. Patients with multimorbidity are more likely to have multidrug treatments; it is, in fact, common in older populations. Patients also may need a longer duration of treatment to get rid of these conditions, which leaves these patients vulnerable to unwanted side effects [2-4]. Multiple studies have already reported that long-term use of drugs has been increasing tremendously [5,6]. Therefore, a growing concern regarding the safety issues associated with long-term prescriptions has recently attracted widespread media attention. Physicians are often asked about the appropriateness of long-term therapy for specific patients.

There has been a significant rise in the number of published studies in which commonly used medications were found to increase or decrease the risk of cancer [7]. Long-term use of insulin seems to be associated with an increased risk of pancreas, liver, kidney, and stomach cancers [8], whereas a protective association was observed between metformin use and colorectal cancer risk in patients with diabetes mellitus [9]. Additionally, antihypertensive drugs are associated with an increased risk of skin cancer [10], but they might have a possible beneficial effect on breast cancer risk [11]. However, there has been substantial controversy about these studies’ validity, and investigations with varying study designs and populations have often arrived at different conclusions. A prudent decision is immediately needed in clinical practices because the use of commonly prescribed medications has been increasing, and the resulting burden of cancer can be substantial at the population level [12].

Big data approaches seem to offer an immense opportunity to generate strong evidence for taking insightful clinical and public health action [13]. Data from electronic medical records and other extended patient registries have been offering expanded research power, especially for analytic studies aiming at association, classification, and prediction [14]. Epidemiological studies have already established a temporal relationship between drugs and diseases and have evaluated a wide range of outcomes. Clinical knowledge is constantly developing as new drug-disease discoveries are made and practices are changed. The knowledge of these associations is valuable but often buried in texts within a range of published literature. Through a web-based approach, information associated with drugs and disease risk can be a great source to understanding the magnitude of the risk between them. The availability of a wide range of associations may be valuable for a variety of applications, including clinical decision support (eg, treatment recommendation), information retrieval, and data mining. We therefore developed a web-based system focusing on the long-term use of commonly prescribed medications, including antihypertensives, antihyperlipidemics, antidiabetics, antihyperuricemics, anxiolytics, hypnotics, sedatives, and nonsteroidal anti-inflammatory drugs (NSAIDs) and 18 different kinds of cancer risk. We developed a system sorted by age, gender, and duration of therapy for exploring cancer risk with commonly used medications.

## Methods

### Ethical Standard

This study is part of a larger project aimed at assessing the effect of most common medications on 20 cancer sites using a population-based nested case-control design. The National Health Insurance Research Database (NHIRD) safeguards the privacy and confidentiality of all beneficiaries and transfers health insurance data to health researchers after ethical approval has been obtained. In this analysis, access to the NHIRD was approved by the Taipei Medical University Joint Institutional Review Board.

### Setting and Data Source

This case-control study was carried out using records from the Taiwan NHIRD, which was established in 1995 and has collects all claims of beneficiaries under the National Health Insurance (NHI) program. The program covers more than 99% of the total population (a total of 23,430,000) and has contracted with 97% of the hospitals and clinics in Taiwan. The NHIRD comprised claims data of 2,000,000 individuals randomly selected from all insured enrollees. This sample represents the original medical claims for all residents in Taiwan covered under the NHI program. The database included specific data on medications prescribed, laboratory and diagnostic test data, dates of visits, lengths of hospitalization, and diagnoses. Diagnoses were coded according to the International Classification of Disease, Ninth Revision, Clinical Modification (ICD-9-CM). Drugs were coded based on the World Health Organization Anatomical Therapeutic Chemical (ATC) classifications (WHO Collaborating Centre for Drug Statistics Methodology ATC/DDD Index). The database used in this study can be interlinked by the scrambled, unique, individual personal identification number.

### Case and Control Selection Criteria

We identified cases in adults who were aged 20 years or older and had received treatment at least two months before the index date. The index date was defined as the date of a cancer diagnosis. We used the ICD-9-CM to identify patients with cancer as cases. Among the NHIRD cases, eligibility criteria for case patients were (1) registration as patients with cancer in the catastrophic illness file, (2) diagnosis of primary cancer in inpatient admission, (3) treatment with any cancer drug from outpatient visits or inpatient admission, (4) a cancer-specific procedure from outpatient visits or inpatient admission, and (5) more than 4 cancer-specific examinations or more than 1 cancer-related procedure (radiotherapy, chemotherapy, or treatment tracking of cancer) from outpatient visits or inpatient admission.

**Figure 1:**
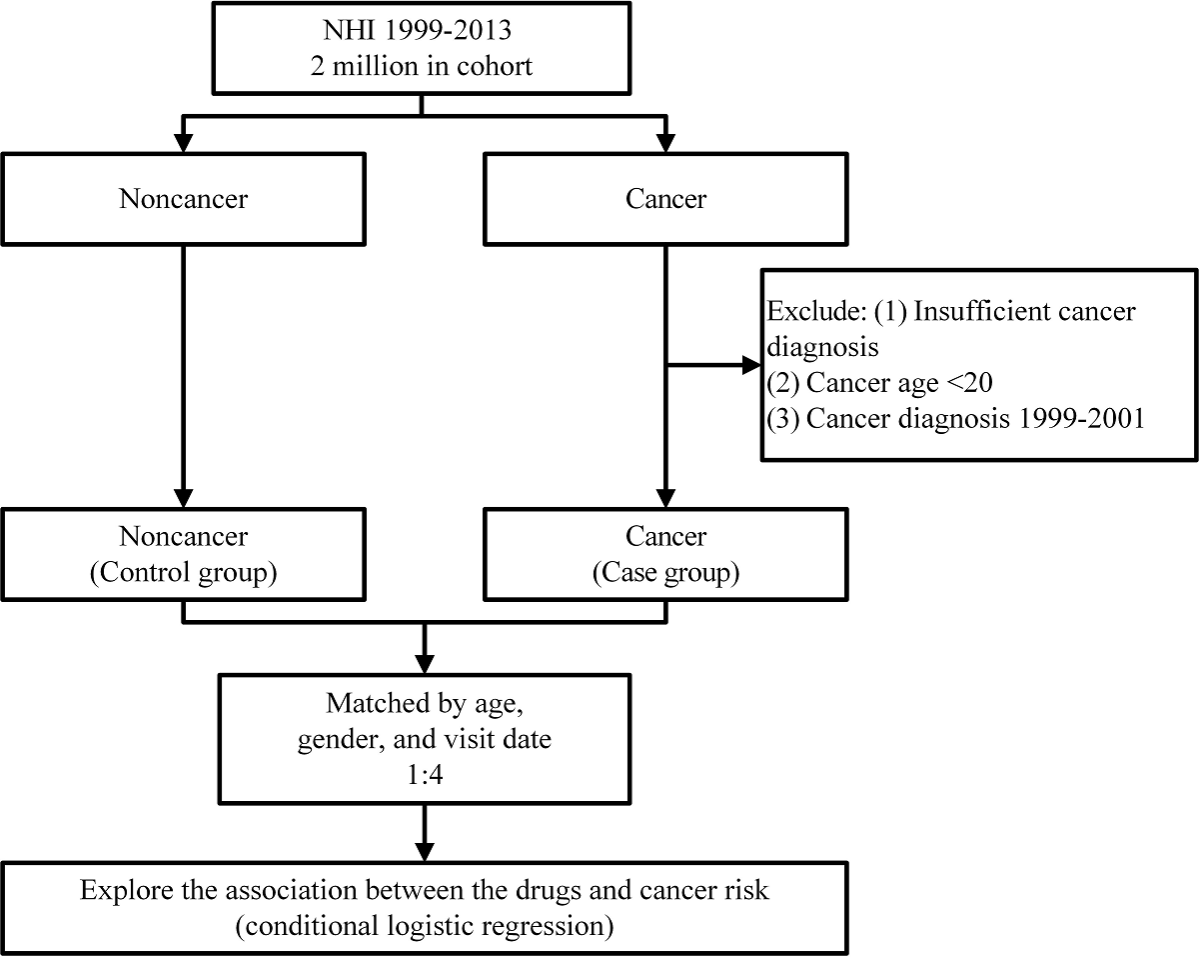
Workflow of the case-control study design. NHI: National Health Insurance.

We randomly selected control patients from patients without a cancer diagnosis during the 15 years (1999-2013) of the study period. Case and control patients were matched 1:4 based on age, sex, and visit date (Figure 1).

### Primary Outcomes

We quantified the risks of common cancers in Taiwan, comparing patients treated with (1) antihypertensives; (2) antihyperlipidemics; (3) antidiabetics; (4) antihyperuricemics; (5) anxiolytics, hypnotics, and sedatives; or (6) NSAIDs against those not prescribed any of these medications. We investigated the following cancers using their corresponding ICD-9-CM codes: oral cancer (140-149.xx, excluding 142.xx and 147.xx), esophageal cancer (150.xx), gastric cancer (151.xx), colorectal cancer (153.xx, rectum 154.xx), liver cancer (155.xx), pancreatic cancer (157.xx), lung cancer (162.xx), skin cancer (172-173.xx), female breast cancer (174.xx), cervical cancer (180.xx), endometrial cancer (182.xx), ovarian cancer (183.xx), prostate cancer (185.xx), bladder cancer (188.xx), kidney cancer (189.xx), brain cancer (191.xx), thyroid cancer (193.xx), non-Hodgkin disease (200.xx, 202.xx, 203.xx), leukemia (204-208.xx), and all cancers (140-208.xx).

### Use of Drugs

**Figure 2:**
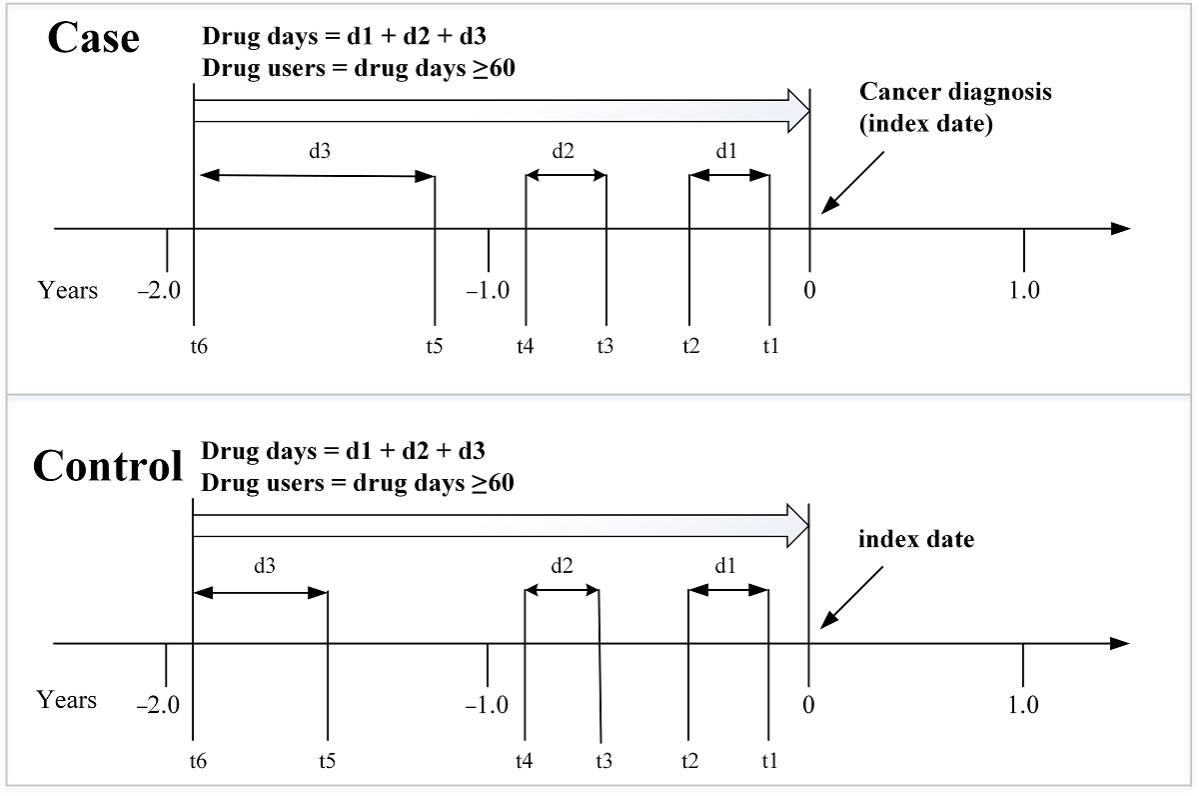
Drugs exposure.

We defined the index date as the date of a cancer diagnosis. The drug exposure was analyzed only before the index date, and we defined drug users as those who filled prescriptions of at least 60 days during admissions and outpatient visits within the 2 years before the index date (Figure 2). This definition was considered for 6 long-term drug groups, namely (1) antihypertensives; (2) antihyperlipidemics; (3) antidiabetics; (4) antihyperuricemics; (5) antianxiety agents, hypnotics, and sedatives; and (6) NSAIDs. Those having no exposure to or receiving these drugs for less than two months were classified as nondrug users.

### Potential Confounders

Comorbidities and medications identified as confounders were adjusted in this study. Comorbidities were defined using the Charlson Comorbidity Index [15], retrieved from outpatient visits before the index date. Any of the comorbidities as confounders were required to be diagnosed at least twice within the 2 years (720 days) before the index date, except for chronic pulmonary disease, which required a diagnosis made more than 4 times. In addition, aspirin (ATC: B01AC06), statins (ATC: C10AA), and metformin (ATC: A10BA2) were also confounders because they might potentially influence the risk of cancers. Exposure to a confounding drug was deﬁned as treatment with the drug for at least 60 days during the 2 years before the index date.

### Statistical Analysis

The McNamara test and paired *t* test were used to test the difference between the case and control groups [16]. Next, conditional logistic regression was conducted to estimate the association between drug exposure and cancer risk by adjusting potential confounders [17]. Table 1 shows our study variables, and conditional logistic regression (temporal model) was adopted to investigate the association between the long-term use of drugs and cancer risk. Age was divided into 4 categories: 20 to 39 years, 40 to 64 years, ≥65 years, and ≥20 years. Gender was classified as male, female, and both. The basic equation of the model was as below, and it may have been slightly modified in different study drug groups.

**Table 1:** Study variables.

Variables	Type	Descriptive	Statistical model	Definition
ID	Nominal	N/A^a^	No	N/A
Age	Ordinal	N/A	No	N/A
Sex	Binomial	1: Yes, 0: No	No	N/A
Study drug (exposure)	Binomial	1: Yes, 0: No	Yes	Independent
Cancer (outcome)	Binomial	1: Yes, 0: No	Yes	Dependent
Myocardial infarction	Binomial	1: Yes, 0: No	Yes	Confounding
Congestive heart failure	Binomial	1: Yes, 0: No	Yes	Confounding
Peripheral vascular disease	Binomial	1: Yes, 0: No	Yes	Confounding
Cerebrovascular disease	Binomial	1: Yes, 0: No	Yes	Confounding
Dementia	Binomial	1: Yes, 0: No	Yes	Confounding
Chronic pulmonary disease	Binomial	1: Yes, 0: No	Yes	Confounding
Rheumatic disease	Binomial	1: Yes, 0: No	Yes	Confounding
Peptic ulcer disease	Binomial	1: Yes, 0: No	Yes	Confounding
Liver disease (mild, moderate, and severe)	Binomial	1: Yes, 0: No	Yes	Confounding
Diabetes (with or without chronic complication)	Binomial	1: Yes, 0: No	Yes	Confounding
Hemiplegia or paraplegia	Binomial	1: Yes, 0: No	Yes	Confounding
Renal disease	Binomial	1: Yes, 0: No	Yes	Confounding
CCI^b^ scores	Ordinal	N/A	Yes	Confounding
Metformin	Binomial	1: Yes, 0: No	Yes	Confounding
Aspirin	Binomial	1: Yes, 0: No	Yes	Confounding
Statin	Binomial	1: Yes, 0: No	Yes	Confounding
Matching number (case match control)	Nominal	N/A	Yes	Stratified

^a^N/A: not applicable.

^b^Charlson Comorbidity Index.

Data analysis and results were performed using SAS software (version 9.4; SAS Institute) [18]. The results were expressed in adjusted odds ratios (AORs), which is e^β1^ with different confidence intervals, like 95%, 99%, and 99.9%. All statistical tests were 2-sided.

### Web-Based System

After analyzing the associations between the long-term use of drugs and cancer risk, we built a web-based system to include all associations [19]. The back end of this web-based system includes a server, database, and application. We used Apache (Apache Software Foundation), MySQL (Oracle Corporation), and PHP (Hypertext Preprocessor) framework for developing the server. Apache is the most commonly used web server software and supports a variety of compiled modules. MySQL is a relational database management system, and PHP is a programming language designed primarily for web development. In this study, we inputted the associations into the database (MySQL) and used PHP to access and process the database. The web-based interface was designed accordingly with HTML, CSS, Bootstrap, JavaScript, and React.

## Results

### Baseline Characteristics

We identified 79,245 participants newly diagnosed with cancer between 2002 and 2013 from 2 million people (Table 2). After matching each case with 4 controls, we included 316,980 matched control patients in this study. The mean age was 59.2 years for both the case and control groups. A slight majority of the participants were male (201,295/396,225, 50.80%), and most participants (202,544/396,225, 51.12%) were aged 40 to 64 years. The prevalence of peptic ulcer disease (12,760/79,245, 16.10% vs 34,283/316,980, 10.82%) and liver disease (11,671/79,245, 14.73% vs 20,647/316,980, 6.51%) in the case group was higher than that in the control group. After associations between long-term use of drugs and cancer risk were comprehensively analyzed and stratified by age and sex, we obtained 45,368 associations in total, of which 2419, 1302, 662, and 366 associations were found statistically significant at a level of *P*<.05, *P*<.01, *P*<.001, and *P*<.0001, respectively (Table S1 in Multimedia Appendix 1).

**Table 2:** Baseline characteristics of case patients and control patients.

Characteristics			Case patients (with cancer) (n=79,245)	Control patients (without cancer) (n=316,980)	*P* value		

**Age (years)**							
	Age (years), mean (SD)	59.20 (15.23)		59.21 (15.24)	N/A^a^		
	20-39, n (%)	8,292 (10.5)		33,168 (10.5)	N/A		
	40-64, n (%)	40,504 (51.1)		162,040 (51.1)	N/A		
	≥65, n (%)	30,449 (38.4)		121,772 (38.4)	N/A		
**Gender, n (%)**							
	Male	40,259 (50.8)		161,036 (50.8)	N/A		
	Female	38,986 (49.2)		155,944 (49.2)	N/A		
**Comorbid conditions, n (%)**							
	Myocardial infarction	372 (0.47)		1729 (0.55)	<.0001		
	Congestive heart failure	2557 (3.23)		9344 (2.95)	<.0001		
	Peripheral vascular disease	1143 (1.44)		4187 (1.32)	<.0001		
	Cerebrovascular disease	5306 (6.70)		22,609 (7.13)	<.0001		
	Dementia	1205 (1.52)		5522 (1.74)	<.0001		
	Chronic pulmonary disease	5951 (7.51)		20,423 (6.44)	<.0001		
	Rheumatic disease	1138 (1.44)		3901 (1.23)	<.0001		
	Peptic ulcer disease	12,760 (16.10)		34,283 (10.82)	<.0001		
	Liver disease	11,671 (14.73)		20,647 (6.51)	<.0001		
	Diabetes	9143 (11.54)		32,532 (10.26)	<.0001		
	Hemiplegia or paraplegia	490 (0.62)		2300 (0.73)	<.0001		
	Renal disease	2793 (3.52)		7453 (2.35)	<.0001		
**Other drugs, n (%)**							
	Metformin	8236 (10.39)		33,375 (10.53)	.07		
	Aspirin	9826 (12.40)		41,726 (13.16)	<.0001		
	Statin	8336 (10.52)		37,395 (11.80)	<.0001		

^a^N/A: not applicable.

### Web-Based System

We successfully developed a web-based system [19], which contains 2 domains: (1) the drug and cancer page and (2) the overview page. The associations between long-term use of drugs and cancer diseases are shown in AORs with 95% CIs. The associations are visualized on the overview page, where researchers can compare and contrast the quantified personalized risk of multiple types of cancers for long-term users of the 6 groups of medications at the same time.

### The Drug and Cancer Association Page

**Figure 3:**
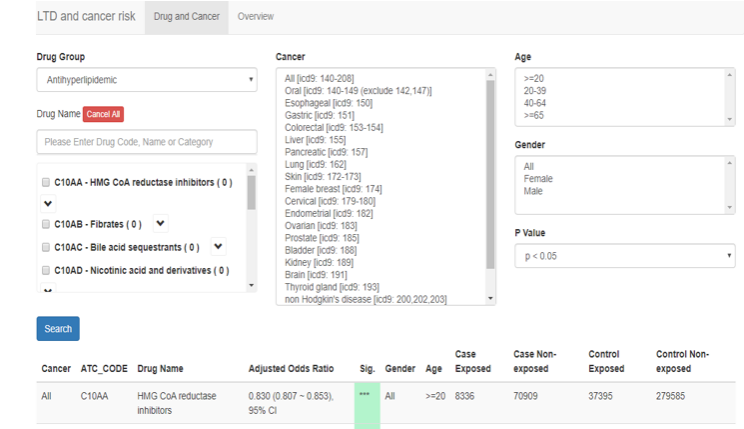
Display of drug and cancer risk.

Drugs were categorized into 6 groups: (1) antihypertensives; (2) antihyperlipidemics; (3) antidiabetics; (4) antihyperuricemics; (5) NSAIDs; and (6) anxiolytics, hypnotics, and sedatives. As exemplified in Figure 3, the web-based system shows an AOR of 0.830 (95% CI 0.807-0.853) between statins (3-hydroxy-3-methyl-glutaryl coenzyme A [HMG-CoA] reductase inhibitors) and the overall cancer risk for those aged 20 years or older. Table 3 shows the associations between different drugs and cancers among different age groups gathered from the developed web-based system.

**Table 3:** Associations between different drugs and cancers among different age groups.

Drug (ATC^a^ code), cancer type, and age (years)				Adjusted odd ratio (95% CI)		Case patients, n				Control patients, n		
						Exposure		Nonexposure		Exposure		Nonexposure
**Aspirin (B01AC06)**												
	**All**											
		≥65	0.954** (0.923-0.985)		6964		23,485		28,665		93,131	
		40-64	0.871*** (0.831-0.913)		2825		37,679		12,925		149,091	
		20-39	1.000 (0.679-1.472)		37		8255		136		33,032	
		≥20	0.924*** (0.900-0.949)		9826		69,419		41,726		275,254	
**Metformin (A10BA02)**												
	**Colorectal**											
		≥65	0.881* (0.794-0.979)		797		4532		3231		18,085	
		40-64	0.799*** (0.701-0.912)		456		4757		2076		18,776	
		20-39	0.448 (0.148-1.358)		4		776		28		3092	
		≥20	0.845*** (0.779-0.916)		1257		10,065		5335		39,953	
**Sitagliptin (A10BH01)**												
	**Pancreatic**											
		≥65	1.901* (1.125-3.213)		28		632		50		2590	
		40-64	2.303* (1.109-4.781)		16		565		27		2297	
		20-39	N/A^b^		0		91		0		364	
		≥20	1.981** (1.298-3.024)		44		1288		77		5251	
**Benzodiazepine derivatives (N05)**												
	**Brain**											
		≥65	1.090 (0.787-1.508)		78		201		256		860	
		40-64	1.456** (1.112-1.905)		111		379		287		1673	
		20-39	2.409** (1.364-4.257)		24		201		46		854	
		≥20	1.379** (1.138-1.670)		213		781		589		3387	

^a^ATC: Anatomical Therapeutic Chemical.

^b^N/A: not applicable.

*P<.05.

**P<.01.

***P<.001.

### The Overview Page

**Figure 4:**
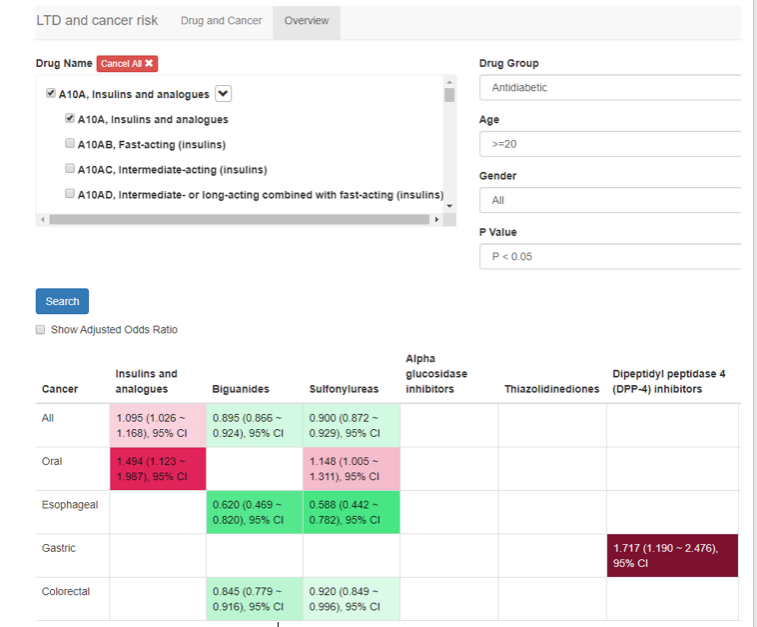
Display of overview.

The web-based system provided an overview of associations between cancers and medications sorted by age, gender, *P* value, and ATC class of medications (Figure 4). In the cells are AORs of each cancer for different medications, and a confidence interval of 95%, 99%, or 99.9% can be selected by users based on different *P* values (*P*<.05, *P*<.01, *P*<.001). The web-based system highlights the cells with different colors—from green to white to red—to demonstrate the direction and the extent of these drug-cancer associations. When a user clicks on the Show Adjusted OR button, the system is able to show all associations. However, if a drug-cancer association pair has a sample size less than 10 patients, the cell stays blank.

Green colors symbolize a significant association between a drug and a cancer with an AOR less than 1. The darker a green color is, the farther from 1 the AOR is. Red colors symbolize a significant association between a drug and a cancer with an AOR greater than 1. The darker a red color is, the farther from 1 the AOR is. White indicates no significant association between a cancer and a drug.

## Discussion

### Main Outcomes

In this nationwide longitudinal retrospective study, we evaluated 79,245 patients with cancer and 316,980 control patients matched by variables including age, sex, and visit date at a 1:4 ratio from the NHIRD in Taiwan, including the follow-up data from 2001 to 2013 of 2 million individuals aged 20 years or older. This web-based system aimed to provide information of medication-cancer associations for users (researchers) to choose potentially clinically relevant ones for further studies (eg, a meta-analysis) and offered a filter by *P* value. We found aspirin and metformin were significantly associated with reduced cancer risk in those aged 40 to 64 years and 65 years or older, but no significant association was uncovered in those aged 20 to 39 years. A partial explanation for this may lie in the fact that the low prescribing rate or the low cancer incidence among those aged 20 to 39 years rendered it impossible for us to reject the null hypothesis that there were no associations between aspirin and all cancers or between metformin and colorectal cancer.

The long-term use of some drugs was associated with increased risk of certain cancers, such as sitagliptin with pancreatic cancer and benzodiazepines (BZDs) with brain cancer. For example, patients aged 40 to 64 years and 65 years or older treated with sitagliptin had a high risk for pancreatic cancer, but there was not sufficient information for us to estimate such risk among patients aged 20 to 39 years. On the contrary, those aged 20 to 39 years receiving BZDs had a higher risk of brain cancer (AOR 2.409, 95% CI 1.364-4.257; *P*=.003) compared with the overall population (AOR 1.379, 95% CI 1.138-1.670; *P*=.001), but there was no significant association between BZDs and brain cancer among those aged 65 years or older.

### Biological Mechanisms

Despite mechanisms between the long-term use of drugs and cancer risk remaining not well understood, our findings were consistent with possible mechanisms proposed in previous studies. Aspirin, metformin, and statins are examples of this. According to previous studies, aspirin reduces prostaglandin generation, which is associated with decreased cellular proliferation, by inhibiting cyclooxygenase isozymes [20]. Metformin activates adenosine monophosphate–activated protein kinase (AMPK), which is a major sensor of whole-body energy metabolism, and activation of AMPK helps to reduce the proliferation of human colon cancer cells [21]. Statins can also decrease intracellular cholesterol production by inhibiting HMG-CoA and may also limit the cellular proliferation required for cancer growth [22]. In this study, aspirin, metformin, and statins were found to have significant associations with overall cancer risk, with AORs of 0.924 (95% CI 0.900-0.949; *P*<.001), 0.845 (95% CI 0.779-0.916; *P*<.001), and 0.830 (95% CI 0.807-0.853; *P*<.001).

Additionally, sitagliptin has been suggested to have an association with elevated risk of pancreatitis and pancreatic cancer [23]. In our results, sitagliptin was also significantly associated with pancreatic cancer risk (AOR 1.981, 95% CI 1.298-3.024; *P*=.002). Another intriguing finding in our study was that cancer risk was significantly associated with angiotensin-converting enzyme inhibitors (ACEIs) (AOR 0.854, 95% CI 0.829-0.880; *P*<.001) but not with angiotensin II antagonists (ARBs), even though both ACEIs and ARBs pharmacologically share a similar pathway. A possible explanation is that the stronger inhibitory effect of ACEIs compared with ARBs on angiotensinogen may be associated with cancer risk. However, our system determined associations only, not causalities, between long-term use of medications and risk of cancers. For some of the significant associations, questions about their mechanisms are still left unanswered. Since the results of this study were associations that did not determine the causality, these associations will need further work to confirm mechanisms and causal relationship between long-term use of drugs and cancer risk.

### Clinical Implication

Despite the immense investment in anticancer therapy, cancer remains the leading cause of death globally [24]. Development of an anticancer drug is resource intensive and takes an average of 13 years at a cost up to US $2.6 billion [25]. The rapidly growing cost and development time have made the pharmaceutical industry a less profitable choice for many investors [26]. Although little attention has been paid to identifying new chemoprevention drugs from existing available drugs, the strategy of using one drug to treat several indications has shown potential success and become an attractive proposition in many areas of medicine, especially in complex disorders [27].

Aspirin is widely used to treat fever and mild pain, but its long-term use may prevent development of squamous cell carcinoma [28], colorectal cancer [29], and hepatocellular carcinoma [30]. Statin use is associated with a reduced risk of pancreatic ductal adenocarcinoma [31] and hepatocellular carcinoma for patients with risk factors [22]. Moreover, metformin, an antidiabetic medication, has drawn attention, since it exhibits an effect on the prevention and treatment of cancers such as colorectal cancer as beneficial as an independent anticancer drug [32]. However, there has been substantial controversy about whether aspirin, statins, and metformin really have anticancer preventive or therapeutic effects on cancers, and often investigations with varying study designs and populations have reached different conclusions. A prudent decision is immediately needed in clinical practices because the use of commonly prescribed medications has been increasing and the resulting burden of cancer can be substantial at the population level [33]. Our study attempted to investigate the magnitude of cancer risk and the benefits of 6 groups of commonly prescribed medications using a large database and appropriate methodology. Our web-based system could potentially show hints of clinical interest for users such as researchers and health care professionals to propose new hypotheses and further undertake research to identify mechanisms or causalities of associations.

### Strengths and Limitations

Strengths of this study include the retrospective study design, long-term follow-up, proper identification of case and control patients, and measurement of the magnitude of association between 6 commonly used groups of medications and cancer risks. Furthermore, confounding factors were appropriately adjusted to reduce the study bias.

We also acknowledge that our research has limitations that need to be addressed. First, drug adherence, self-payment, laboratory data, and lifestyles characteristics such as body mass index, smoking, and family history of cancer were unavailable in the NHIRD. Second, other risk factors for cancer, such as phenotype, genotype, and exposure type, might have influenced the results. Although we applied the match method and adjustment for numerous covariates to control confounding factors, it was impossible to eliminate all confounding factors, particularly indications. Third, all data were collected from the Taiwan NHIRD, and hence, the study population limited the generalization of the results to other countries with different ethnic distribution. Fourth, the results showed associations between the long-term use of drugs and cancer risk but not causation.

Moreover, we did not set a threshold for statistical significance at 0.05/45,368 ≈ 1.10 × 10^–6^ for multiple testing correction, given the large number of statistical tests and the highly selected patients—patients with cancer and long-term users of medications instead of the general population. Had we set the significance level at 1.10 × 10^–6^, there would not have been enough significant associations to be useful or practical to users. Therefore, we offered in the web-based system a filter by *P* value, allowing users to choose a *P* value based on their own need for research. Moreover, considering that there might have been a small number of these highly selected patients, especially after we grouped by drug class, cancer type, age, and gender, we provided users with detailed information of sample sizes on the web-based system, showing the numbers of case and control patients either exposed or not exposed to the study medications.

### Conclusion

This comprehensive retrospective study not only provides an overview of associations of cancer risk with 6 commonly prescribed groups of medications but also helps to narrow the gap in the currently insufficient research on the long-term safety of these medications. With all the quantified data visualized, the system is expected to further facilitate research on cancer risk and prevention. Since our findings have proposed only associations between cancers and long-term use of medications, further clinical trials and meta-analyses are required to assess and confirm their causality. This web-based system could potentially serve as a stepping-stone to exploring and consulting associations between long-term use of drugs and cancer risk.

## Appendix

Multimedia Appendix 1 Supplementary table.

## Abbreviations

ACEI: angiotensin-converting enzyme inhibitors
AMPK: adenosine monophosphate–activated protein kinase
AOR: adjusted odds ratio
ARB: angiotensin II antagonist
ATC: Anatomical Therapeutic Chemical
BZD: benzodiazepine
HMG-CoA: 3-hydroxy-3-methyl-glutaryl coenzyme A
ICD-9-CM: International Classification of Disease, Ninth Revision, Clinical Modification
NHI: National Health Insurance
NHIRD: National Health Insurance Research Database
NSAID: nonsteroidal anti-inflammatory drug
PHP: Hypertext Preprocessor
