# Supplementary material for: Development of a Web-Based System for Exploring Cancer Risk With Long-term Use of Drugs: Logistic Regression Approach
Source: JMIR Public Health Surveill. 2021 Feb 15;7(2):e21401. doi: 10.2196/21401 (PMC7920756; doi:10.2196/21401)
Supplement: Multimedia Appendix 1 [file publichealth_v7i2e21401_app1.docx]

**Supplementary Table 1.** Number of significant associations at different significance levels.

| **No. of associations** | | **Significance level**  No. of significant associations (%) | | | | | | | |
| --- | --- | --- | --- | --- | --- | --- | --- | --- | --- |
|  |  | *p*<0.05 | | *p*<0.01 | | *p*<0.001 | | *p*<0.0001 | |
| **Total** | **45,368** | **2,419** | **(5.3)** | **1,302** | **(2.9)** | **662** | **(1.5)** | **366** | **(0.8)** |
| “All cancers” | 2,568 | 535 | (20.8) | 363 | (14.1) | 241 | (9.4) | 171 | (6.7) |
| Non-sex-specific 15 cancers | 38,520 | 1,661 | (4.3) | 827 | (2.1) | 382 | (1.0) | 175 | (0.5) |
| Sex-specific cancers |  |  |  |  |  |  |  |  |  |
| Cervical | 856 | 20 | (2.3) | 7 | (0.8) | 1 | (0.1) | 0 | (0.0) |
| Endometrial | 856 | 30 | (3.5) | 10 | (1.2) | 2 | (0.2) | 0 | (0.0) |
| Female breast | 856 | 76 | (8.9) | 38 | (4.4) | 15 | (1.8) | 4 | (0.5) |
| Ovarian | 856 | 24 | (2.8) | 12 | (1.4) | 2 | (0.2) | 0 | (0.0) |
| Prostate | 856 | 73 | (8.5) | 45 | (5.3) | 19 | (2.2) | 16 | (1.9) |
